# Divergent immune landscapes of primary and syngeneic Kras-driven mouse tumor models

**DOI:** 10.1038/s41598-020-80216-1

**Published:** 2021-01-13

**Authors:** Wade R. Gutierrez, Amanda Scherer, Gavin R. McGivney, Qierra R. Brockman, Vickie Knepper-Adrian, Emily A. Laverty, Grace A. Roughton, Rebecca D. Dodd

**Affiliations:** 1 Cancer Biology Graduate Program, Carver College of Medicine, University of Iowa, 285 Newton Rd, 3269C CBRB, Iowa City, IA 52246 USA; 2 Medical Scientist Training Program, University of Iowa, Iowa City, IA USA; 3 Holden Comprehensive Cancer Center, University of Iowa, Iowa City, IA USA; 4 Molecular Medicine Graduate Program, University of Iowa, Iowa City, IA USA; 5 Department of Internal Medicine, University of Iowa, Iowa City, IA USA

**Keywords:** Cancer models, Sarcoma, Cancer microenvironment, Tumour immunology

## Abstract

Immune cells play critical functions in cancer, and mice with intact immune systems are vital to understanding tumor immunology. Both genetically engineered mouse models (GEMMs) and syngeneic cell transplant approaches use immunocompetent mice to define immune-dependent events in tumor development and progression. Due to their rapid and reproducible nature, there is expanded interest in developing new syngeneic tools from established primary tumor models. However, few studies have examined the extent that syngeneic tumors reflect the immune profile of their originating primary models. Here, we describe comprehensive immunophenotyping of two well-established GEMMs and four new syngeneic models derived from these parental primary tumors. To our knowledge, this is the first systematic analysis comparing immune landscapes between primary and orthotopic syngeneic tumors. These models all use the same well-defined human-relevant driver mutations, arise at identical orthotopic locations, and are generated in mice of the same background strain. This allows for a direct and focused comparison of tumor immune landscapes in carefully controlled mouse models. We identify key differences between the immune infiltrate of GEMM models and their corresponding syngeneic tumors. Most notable is the divergence of T cell populations, with different proportions of CD8+ T cells and regulatory T cells across several models. We also observe immune variation across syngeneic tumors derived from the same primary model. These findings highlight the importance of immune variance across mouse modeling approaches, which has strong implications for the design of rigorous and reproducible translational studies.

## Introduction

Immune cells play critical roles in cancer by impacting tumor growth, therapeutic response, and metastatic progression. Immunocompetent mouse models are vital tools for understanding the role of the immune system in tumor biology. Two approaches that utilize immunocompetent hosts are genetically-engineered mouse models (GEMMs) and syngeneic tumor models^1,2^. In GEMMs, primary tumors are generated de novo in genetically modified mice that contain activatable alleles of common cancer mutations. This approach can produce tumors with a high-fidelity to human cancer, but GEMMs often require large numbers of mice and extended latency periods. In contrast, syngeneic models (allografts) are generated by implanting murine cancer cells into mice of the same background strain as the original tumor. Syngeneic approaches facilitate rapid and reproducible preclinical screens due to shorter latency periods, uniform tumor growth, and ease of experimental manipulation. Both GEMM and syngeneic approaches allow for tumors to arise at orthotopic locations, which is important for establishing a tumor microenvironment similar to patient tumors.

Tumor immune profiles are dependent on genetic mutations, murine background, cancer type, and tumor location. Genetic mutations can drive different patterns of cancer immunity. For example, loss of PTEN in melanoma models reduces CD8 + T cell infiltration and promotes resistance to T cell-mediated therapy^3^. Mouse background strain modifies the immune landscape of GEMMs, as altered levels of myeloid and lymphoid infiltration are observed in genetically-matched tumors from BALB/c and C57BL/6 mice^4^. Among syngeneic tumors, both cancer type and mouse strain determine the composition of immune infiltrate^5–9^. Across BALB/c syngeneic models, myeloid derived suppressor cells (MDSCs) are the most abundant immune cells in 4T1 breast tumors, while macrophages are dominant in RENCA kidney tumors, and NK cells comprise the majority of immune cells in CT26 colorectal cancers^5^. Cancer type also impacts the level of total immune infiltration in syngeneic models, which can range from immune-rich (~ 40% live cells in RENCA tumors) to immune-poor (~ 4% live cells in B16F10 melanoma tumors)^6^. Even among the same syngeneic model, tumor location can alter the immune profile, as orthotopically injected CT26 tumors contain higher levels of T, B, and NK cells than tumors grown subcutaneously^10^. The tumor immune landscape is also an important determinant of therapeutic outcomes, including responses to cytotoxic, targeted, and immune therapy approaches^5,7,8,11–14^. Therefore, a better understanding of immune infiltrates between primary GEMM tumors and their syngeneic counterparts is critical for design and interpretation of cancer immunology studies. While many studies use syngeneic and GEMM models interchangeably, few have examined how well syngeneic tumors reflect the immune profile of their originating primary models. Here, we report the first comprehensive analysis of immune landscapes between primary and orthotopic syngeneic models using two well-established soft-tissue sarcoma GEMMs and four new syngeneic models derived from these primary tumors. Importantly, all tumor models used in this study contain identical initiating mutations and arise at similar orthotopic locations in the same background strain of mice, allowing for a direct and focused comparison of carefully controlled tumor immune landscapes.

Soft-tissue sarcomas (STS) are aggressive mesenchymal tumors of the connective tissue that arise in a diverse array of tissues, including the muscle. There are over 50 different subtypes of STS, defined by distinct tissue histologies, genetic profiles, and tissues of origin. Ras pathway activation is common in sarcomas and occurs in two of the most aggressive forms of STS: Undifferentiated Pleomorphic Sarcoma (UPS) and Rhabdomyosarcoma (RMS)^15–18^. UPS originates in skeletal muscle and is one of the most common STS in adult patients. RMS arises from muscle stem cells (called satellite cells) and is the most common STS in pediatric patients. Both UPS and RMS harbor similar genetic alterations, including activation of the Ras pathway and disruptions in common tumor suppressors, such as p53^18–20^. High-fidelity GEMM models of UPS and RMS have been successfully used in determining mechanisms of disease and identifying new treatment paradigms for these aggressive tumors^21–26^. Both UPS and RMS GEMM approaches utilize localized Cre activation to drive activation of oncogenic Kras and loss of p53 in LSL-Kras^G12D^; p53^Flox/Flox^ (KP) mice. These mice develop high-grade tumors that resemble human sarcomas at the molecular, histologic, and physiologic levels^27–30^. For UPS models, adenovirus expressing Cre recombinase (Ad-Cre) is injected directly into the gastrocnemius muscle of KP mice to initiate tumors at the site of Ad-Cre injection. RMS is generated in Pax7-CreER; KP mice that restricts tamoxifen-regulated Cre activity to Pax7-expressing satellite cells. Localized injection of tamoxifen (TMX) into the gastrocnemius activates Cre, resulting in Kras^G12D^ activation and p53 loss in muscle satellite cells. Importantly, both UPS and RMS tumors arise within a native tumor microenvironment, facilitating studies on immune infiltration and treatment response^31^.

Because both GEMM approaches use identical initiating mutations in mice of the same background at similar orthotopic locations, these models provide a unique opportunity to directly compare the immune profiles of different preclinical tumor models in genetically matched tumors. Using comprehensive immunophenotyping of UPS and RMS primary GEMMs, we identified significant divergence of the T cell populations between the two models. To determine if the immune landscapes of syngeneic tumors reflect the profiles of their primary counterparts, we generated four new syngeneic sarcoma models from orthotopically-injected cells named K-Ras Induced Murine Sarcoma (KRIMS) tumors. While these cell transplant models have similar histology and growth kinetics as their primary tumor counterparts, they are highly divergent in their immune landscapes, particularly in the T cell compartments of certain syngeneic models. Importantly, immune profiles differ between syngeneic tumors derived from the same GEMM, demonstrating the variability of immune phenotypes among genetically-related models. This study provides the first systematic characterization of intratumoral immune profiles between paired primary and syngeneic tumor models, which has strong implications for the design of preclinical studies using murine approaches.

## Results

### Tumors from UPS and RMS GEMMs have divergent immune profiles

**Figure 1:**
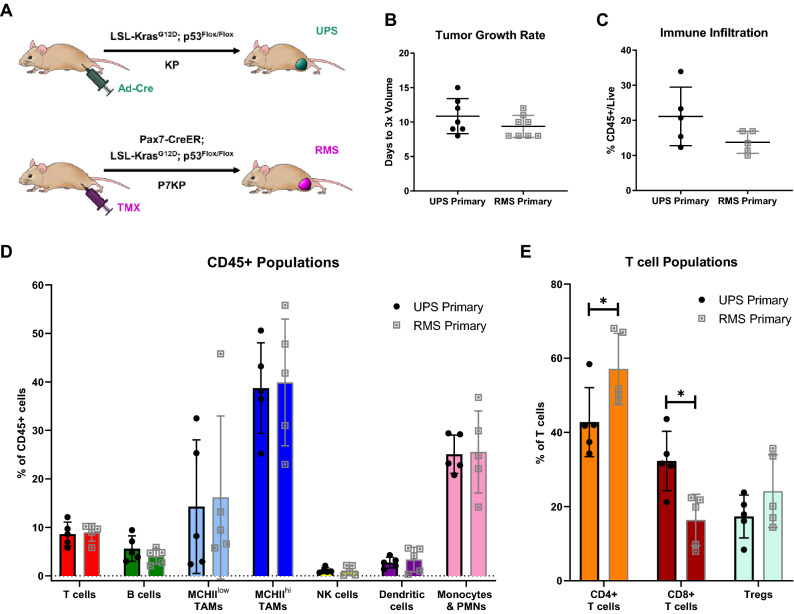
Primary UPS and RMS tumors differ in initiation time and intratumoral T cell populations. (**A**) Generation of UPS and RMS primary tumors. Primary UPS tumors are induced in LSL-Kras^G12D^, p53^Flox/Flox^ (KP) mice by intramuscular injection of Ad-Cre to locally delete p53 and activate oncogenic Kras. Primary RMS tumors are induced in Pax7-CreER; KP (P7KP) mice by intramuscular injection of tamoxifen (TMX) to activate Cre in Pax7-expressing satellite cells, resulting in deletion of p53 and activation of oncogenic Kras in muscle stem cells. (**B**) Primary UPS and RMS tumors proliferate at similar rates, tripling in volume approximately 10 days after detection. (**C**) Level of total immune cell infiltration (CD45 + cells) is similar between primary UPS and RMS tumors. (**D**) Comparable levels of T cells, B cells, tumor-associated macrophages (TAMs), NK cells, dendritic cells, and monocytes/PMNs are observed between the two models. (**E**) The T cell compartments display significant differences between the two models. Primary UPS tumors contain less CD4 + T cells (% of CD3 + T cells) and more CD8 + T cells (% of CD3 + T cells) than primary RMS tumors. Levels of Tregs (% of CD4 + T cells) are similar between the two models. Unpaired t tests with Welch’s correction were used to analyze (**B**,**C**) and individual populations in (**D**,**E**). Data represent individual tumors with the mean ± SD. A *p*-value < 0.05 is denoted by a star. *n* = 7 tumors for growth data (**B**) and *n* = 5 tumors for immune analysis (**C**–**E**).

Both the UPS and RMS models are muscle-resident tumors with identical initiating mutations in the same murine background. However, they differ in sarcoma type and tumor onset due to different cells of origin and Cre activation strategies (Fig. 1A)^27–30^. Similar to published data, UPS tumors in this study develop 12.4 weeks after Ad-Cre injection (range 9–17 weeks), and RMS tumors develop 6.0 weeks after TMX injection (range 5–7 weeks) (Supplemental Fig. 1A). Proliferative rates are similar between the two models, and are reported as the number of days required to triple in volume (Fig. 1B). UPS primary tumors triple in 10.9 days (range 8 to 15 days), with RMS primary tumors proliferating at a similar rate of 9.4 days to tripling in volume (range 8 to 12 days).

To determine how sarcoma type impacts the immune profile of GEMM tumors, we performed extensive flow cytometry-based immunoprofiling of each primary tumor model. Average levels of total immune cell infiltration (CD45 +) are similar across both models, comprising 14% and 21% of all live cells in RMS and UPS tumors, respectively (Fig. 1C). Next, we performed comprehensive immunophenotyping of ten innate and adaptive cell populations: B cells, dendritic cells, natural killer (NK) cells, monocytes/PMNs (polymorphonuclear neutrophils), tumor-associated macrophages (TAMs, including MHCII^low^ and MHCII^hi^ populations), and T cell subsets (total CD3 + T cells, CD4 + T cells, CD8 + T cells, and Tregs) (Fig. 1D). Myeloid cells are the dominant immune population in both tumor types, with TAMs and monocytes/PMNs comprising up to 80% of all immune cells. Both UPS and RMS primary tumors have similar amounts of myeloid cell infiltration. Average levels of tumor-infiltrating B cells, NK cells and dendritic cells are also comparable between UPS and RMS tumors. Both dendritic and B cells represent 2–6% of total immune cells, with NK cells comprising < 2% of tumor-resident immune cells. Total CD3 + T cells are similar between UPS and RMS primary models, comprising 8–10% of total immune cells. However, the compositions of the T cell compartments are considerably different between the two models (Fig. 1E). Levels of cytotoxic CD8 + T cells are double in UPS tumors compared with RMS primary tumors (32% vs. 16%). In contrast, CD4 + T cells are enriched in RMS tumors compared to UPS (57% vs. 42%). These findings highlight T cell-specific changes in the tumor immune landscape that is dependent on sarcoma type rather than initiating mutations.

### Generation of new syngeneic sarcoma models

**Figure 2:**
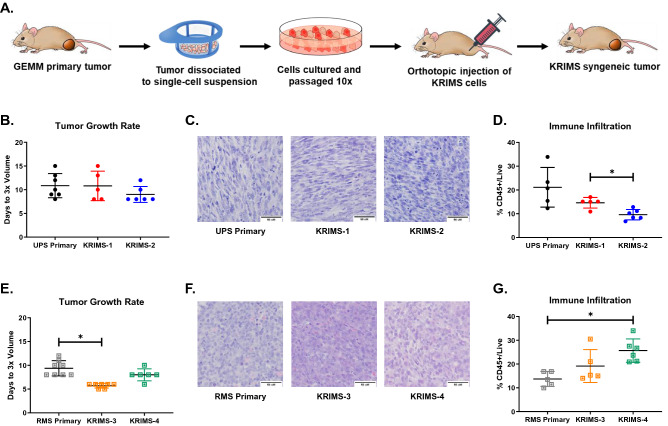
Development of orthotopic syngeneic models from primary UPS and RMS tumors. (**A**) Generation of syngeneic models. Expression of oncogenic Kras^G12D^ and deletion of p53 drives tumor development in GEMMs. After tumors harvest, cells are dissociated into a single cell suspension. Kras Induced Murine Sarcoma (KRIMS) cell lines are generated following 10 passages. KRIMS cells are injected orthotopically into immunocompetent mice to form syngeneic tumors. (**B**–**D**) Characterization of UPS syngeneic models, KRIMS-1 and KRIMS-2. (**B**) Proliferative rates are similar between primary and syngeneic UPS models, as determined by the days required for tumors to triple in volume. (**C**) All UPS models display similar pathological features, including enlarged nuclei and spindle cell morphology. Images at 40x, scale bar at 50 µm. (**D**) Syngeneic UPS tumors contain less immune cell infiltration than primary UPS tumors, determined by flow cytometry for CD45 + cells. (**E**–**G**) Characterization of RMS syngeneic models, KRIMS-3 and KRIMS-4. (**E**) KRIMS-3 syngeneic tumors have a modest growth advantage compared to primary RMS tumors, while KRIMS-4 tumors grow at a similar rate. Growth rates are reported as the time required for tumors to triple in volume. (**F**) Primary and syngeneic RMS models display hallmark features of RMS pathology, including small, round cells. Images at 40x, scale bar at 50 µm. (**G**) The syngeneic RMS model KRIMS-4 displays higher immune cell infiltration than primary RMS tumors, determined by flow cytometry for CD45 + cells. Welch’s ANOVA and Dunnett’s T3 multiple comparison test were used to analyze data in (**B**,**D**,**E**,**G**). Data represent individual tumors with the mean ± SD. A *p*-value < 0.05 is denoted by a star. *n* = 5–7 tumors for growth data (**B**,**E**) and *n* = 5–6 tumors for immune analysis (**D**,**G**).

Primary tumor models are a cornerstone of cancer biology, but cell transplant approaches offer several advantages, including accelerated tumor initiation, increased reproducibility, and reduced cost. To develop orthotopic syngeneic models, we generated UPS and RMS cell lines by enzymatic digestion of primary tumors (Fig. 2A). We named these cell series KRIMS for K-Ras Induced Murine Sarcomas. Similar to other syngeneic cell series, this collection can be used to compare tumor phenotypes across different sarcoma types. We generated four new orthotopic syngeneic models: UPS models are derived from KRIMS-1 and KRIMS-2 cells, while RMS tumors develop from KRIMS-3 and KRIMS-4 cells. All tumors developed 1 to 3 weeks after injection (Supplemental Fig. 1B). Tumor tripling data shows that growth rates of KRIMS-1 and KRIMS-2 tumors are similar to UPS primary tumors (Fig. 2B and Supplemental Fig. 1C). Histological analysis confirms syngeneic UPS models display pathological features similar to the primary model, including multiple mitoses, enlarged nuclei, and spindle cell morphology (Fig. 2C). The syngeneic RMS models, KRIMS-3 and KRIMS-4, display comparable growth kinetics to primary RMS tumors, with a slight growth acceleration of KRIMS-3 tumors (Fig. 2E and Supplemental Fig. 1D). Syngeneic RMS tumors contain hallmark features of RMS pathology, including small round cells with hypochromic nuclei and eosinophilic cytoplasm (Fig. 2F). While the KRIMS cells display variable growth rates in vitro, (Supplemental Fig. 2), these do not translate into substantial growth differences in vivo. Taken together, these data demonstrate that the new syngeneic models closely resemble key features of established primary tumor models.

### Immune infiltration of syngeneic models differs from primary GEMM tumors

Few studies have compared the immune profiles of orthotopic allograft tumors with their originating primary model to determine how accurately syngeneic tumors recapitulate the immune landscapes of their corresponding GEMMs. First, we examined levels of total immune infiltrate between primary and syngeneic tumors of each sarcoma type. Across the UPS models, syngeneic tumors have less immune infiltration than primary tumors (Fig. 2D). For example, the number of CD45 + cells in primary UPS tumors is double the number found in KRIMS-2 tumors (21% vs. 10%, *p* = 0.0976). In the RMS models, syngeneic tumors contain more immune cells than primary tumors, with KRIMS-4 allografts containing almost twice the amount of immune infiltrate than primary tumors (26% vs. 14%, *p* = 0.0025) (Fig. 2G). These results suggest that levels of immune infiltration can differ between primary and syngeneic models, and even between individual models within a syngeneic series.

### Immune landscapes of UPS primary and syngeneic models are highly divergent

**Figure 3:**
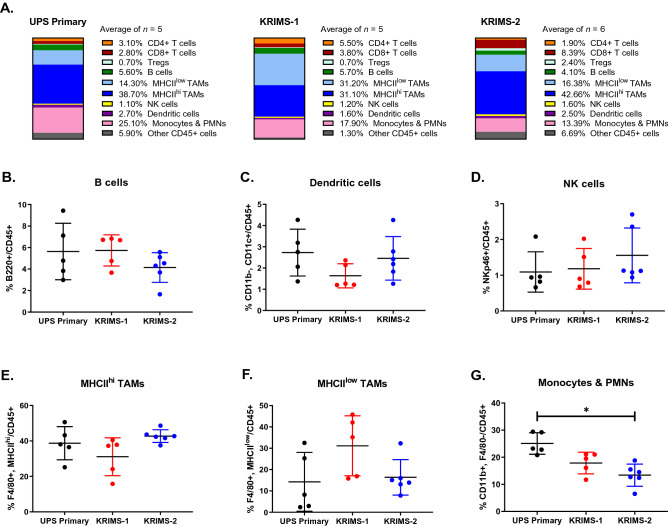
The tumor immune landscapes of UPS primary and syngeneic tumors. (**A**) Average frequencies of immune cell populations in UPS primary and syngeneic models, reported as percentages of CD45 + cells. Mean values are calculated from all individual tumors from this analysis. (**B**–**G**) Flow cytometry analysis of immune cell populations across individual tumors identifies similarities in myeloid populations between UPS primary and syngeneic tumors. Immune cell populations include (**B**) B cells (B220 +), (**C**) dendritic cells (CD11b-, CD11c +), (**D**) natural killer cells (NKp46 +), (**E**) MHCII^hi^ tumor-associate macrophages (F4/80 + , MHCII^hi^), (**F**) MHCII^low^ tumor-associated macrophages (F4/80 + , MHCII^low^), and (**G**) monocytes/PMNs (CD11b + , F4/80-). KRIMS-2 tumors have lower levels of monocytes/PMNs compared to the UPS primary tumors. Welch’s ANOVA and Dunnett’s T3 multiple comparison test were used to analyze data in (**B**–**G**). Data represent mean values in (**A**) and individual tumors with the mean ± SD in (**B**–**G**). A *p*-value < 0.05 is denoted by a star. *n* = 5–6 tumors for immune analysis (**A**–**G**).

Next, we examined the composition of the UPS immune landscape between primary and syngeneic models (Fig. 3A and Supplemental Fig. 3). When viewing immune profiles as a snapshot of the total immune infiltrate, this analysis illustrates an overall similarity between primary UPS, KRIMS-1 and KRIMS-2 tumors. TAM and monocytes/PMN populations, which include MDSCs, comprise the largest proportion of immune cells in all models (blue and pink bars, respectively). Next, we evaluated the proportion of each cell type across individual tumors. We observe similar levels of B cells, dendritic cells, and NK cells across all three models (Fig. 3B–D). As TAMs account for the largest proportion of tumor-resident immune cells, we sub-divided this population into MHCII^hi^ and MHCII^low^ populations. MHCII^hi^ TAMs are mature, tissue-resident macrophages, while MHCII^low^ TAMs represent a phenotypically distinct macrophage population linked to tumor progression^32–34^. We found that levels of MHCII^hi^ and MHCII^low^ TAMs are similar between UPS primary and syngeneic tumors (Fig. 3E–F). MHCII^hi^ TAMs are more prevalent than MHCII^low^ TAMs in all models, as shown by an MHCII^hi^/MHCII^low^ ratio > 1 (Supplemental Fig. 4A). Monocytes/PMNs are more abundant in UPS primary tumors than in syngeneic models, comprising twice the number of cells in primary than KRIMS-2 tumors (25% vs. 13%, *p* = 0.0028) (Fig. 3G).

**Figure 4:**
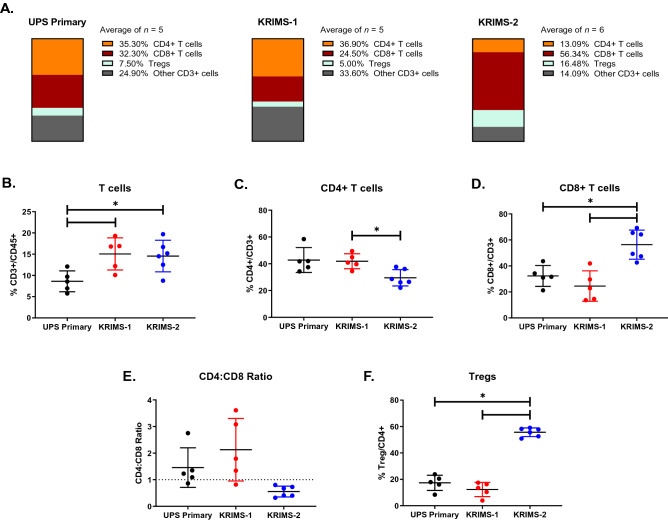
Variability across T cell compartments in UPS primary and syngeneic tumors. (**A**) Average frequencies of T-cell populations in UPS primary and syngeneic models, reported as percentages of total CD3 + T cells. Mean values are calculated from all individual tumors from this analysis. (**B**–**F**) Flow cytometry analysis of immune cell populations across individual tumors identifies divergence in T cell compartments between UPS primary and syngeneic tumors. T cell populations include (**B**) CD3 + T cells, (**C**) CD4 + T cells, (**D**) CD8 + T cells, (**E**) CD4:CD8 ratio (dotted line denotes a ratio equal to 1) and (**F**) regulatory T cells (Tregs). Infiltration of CD3 + T cells is higher in syngeneic tumors than in primary UPS tumors. In comparison to UPS primary and KRIMS-1 tumors, KRIMS-2 tumors have decreased levels of CD4 + T cells and increased levels of Tregs and CD8 + T cells. Welch’s ANOVA and Dunnett’s T3 multiple comparison test were used to analyze data in (**B**–**F**). Data represent mean values in (**A**) and individual tumors with the mean ± SD in (**B**–**F**). A *p*-value < 0.05 is denoted by a star. *n* = 5–6 tumors for immune analysis (**A**–**F**).

The T cell compartment displays extensive differences between UPS primary and syngeneic tumors (Fig. 4A). Levels of total T cell infiltration are almost twice as high in KRIMS-1 and KRIMS-2 tumors as in UPS primary tumors (15% vs. 8%) (Fig. 4B). Within the T cell compartment, levels of CD4 + T cells, CD8 + T cells, and Tregs vary greatly across tumor models, with KRIMS-2 tumors showing high divergence from UPS primary and KRIMS-1 tumors (Fig. 4C–F). CD8 + T cells are nearly doubled in KRIMS-2 tumors, representing 56% of total T cells vs. ~ 25–32% in the other models (Fig. 4D). Conversely, infiltration of CD4 + T cell populations into KRIMS-2 tumors is lower than into KRIMS-1 tumors. The CD4:CD8 ratio further illustrates these T cell-specific phenotypes (Fig. 4E), which shows CD4 + T cells are more prominent in primary and KRIMS-1 tumors (ratio > 1), while CD8 + T cells are dominant in KRIMS-2 tumors (ratio < 1). Treg populations are also highly variable across the models, with KRIMS-2 tumors containing three times more Tregs than in either UPS primary or KRIMS-1 tumors (Fig. 4F). Together, these data identify strong differences in the immune composition of primary and certain syngeneic UPS models, particularly in T cell subsets.

### Diverse T cell landscapes across RMS primary and syngeneic models

**Figure 5:**
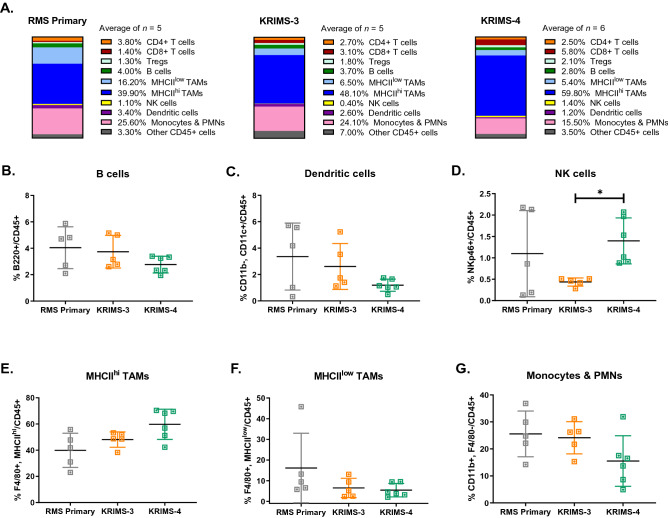
RMS primary and syngeneic tumor immune profiles. (**A**) Average frequencies of immune cell populations in RMS primary and syngeneic models, reported as percentages of CD45 + cells. Mean values are calculated from all individual tumors from this analysis. (**B**–**G**) Flow cytometry analysis of immune cell populations across individual tumors identifies similarities in myeloid populations between RMS primary and syngeneic tumors. Immune cell populations include (**B**) B cells (B220 +), (**C**) dendritic cells (CD11b-, CD11c +), (**D**) natural killer cells (NKp46 +), (**E**) MHCII^hi^ tumor-associate macrophages (F4/80 + , MHCII^hi^), (**F**) MHCII^low^ tumor-associated macrophages (F4/80 + , MHCII^low^), and (**G**) monocytes/PMNs (CD11b + , F4/80-). KRIMS-4 tumors contain more NK cells than KRIMS-3 tumors. No other differences in myeloid populations were observed. Welch’s ANOVA and Dunnett’s T3 multiple comparison test were used to analyze data in (**B**–**G**). Data represent mean values in (**A**) and individual tumors with the mean ± SD in (**B**–**G**). A *p*-value < 0.05 is denoted by a star. *n* = 5–6 tumors for immune analysis (**A**–**G**).

The intratumoral immune profiles of RMS models display several parallels to UPS tumors. Similar to UPS models, myeloid cells remain the dominant immune population in RMS primary, KRIMS-3, and KRIMS-4 tumors (Fig. 5A and Supplemental Fig. 3B), representing ~ 80% of all immune cells. Levels of TAMs and monocytes/PMNs are similar across primary, KRIMS-3, and KRIMS-4 models (Fig. 5G). In all RMS models, MHCII^hi^ TAMs are more prevalent than MHCII^low^ TAMs (Supplemental Fig. 4B). Indeed, MHCII^hi^ TAMs are enriched 16-fold compared to MHCII^low^ TAMs in KRIMS-4 tumors, representing 60% vs. 5% of total CD45 + cells (Fig. 5E–F and Supplemental Fig. 3C). Of note, the syngeneic RMS models have the lowest levels of MHCII^low^ TAMs across all tumors we analyzed, which gives the syngeneic RMS models much higher MHCII^hi^/MHCII^low^ ratios than syngeneic UPS models (Supplemental Fig. 4). Both B cells and dendritic cells are comparable across all RMS models, each representing 1–6% of the immune infiltrate (Fig. 5B–C). Levels of NK cells are similar between primary and syngeneic tumors, although KRIMS-4 tumors contain more NK cells than KRIMS-3 tumors (Fig. 5D).

**Figure 6:**
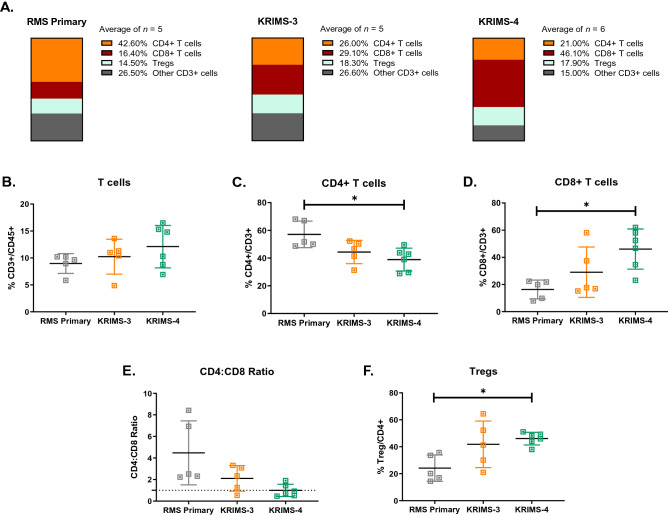
Divergence of T cell populations between primary and syngeneic RMS models. (**A**) Average frequencies of T-cell populations in UPS primary and syngeneic models, reported as percentages of total CD3 + T cells. Mean values are calculated from all individual tumors from this analysis. (**B**–**F**) Flow cytometry analysis of immune cell populations across individual tumors identifies divergence in T cell compartments between RMS primary and syngeneic tumors. T cell populations include (**B**) CD3 + T cells, (**C**) CD4 + T cells, (**D**) CD8 + T cells, (**E**) CD4:CD8 ratio (dotted line denotes a ratio equal to 1) and (**F**) regulatory T cells (Tregs). The KRIMS-4 syngeneic models have lower levels of CD4 + T cell infiltration and elevated amounts of Tregs and CD8 + T cells compared to primary RMS tumors. Welch’s ANOVA and Dunnett’s T3 multiple comparison test were used to analyze data in (**B**–**F**). Data represent mean values in (**A**) and individual tumors with the mean ± SD in (**B**–**F**). A *p*-value < 0.05 is denoted by a star. *n* = 5–6 tumors for immune analysis (**A**–**F**).

Similar to UPS models, the T cell populations of RMS models are highly divergent (Fig. 6A). While levels of total T cell infiltration are similar between RMS primary and syngeneic tumors (Fig. 6B), the composition of T cell compartments is highly variable (Fig. 6A). KRIMS-4 tumors contain over twice as many CD8 + T cells as primary RMS tumors (46% vs. 16%). Levels of Tregs are also doubled in KRIMS-4 tumors compared to primary tumors (46% vs. 24%). In contrast, CD4 + T cells are decreased in KRIMS-4 tumors compared to RMS primary tumors (Fig. 6C–F). Similar elevation of CD8 + and Tregs are observed in KRIMS-3 tumors, but these trends are not statistically significant. These data lead to an inverted CD4:CD8 ratio across the RMS models, with primary and KRIMS-3 tumors containing more CD4 + T cells than CD8 + T cells (ratio > 1), and KRIMS-4 tumors containing similar amounts of CD4 + T cells and CD8 + T cells (ratio = 0.9933) (Fig. 6E). These findings from RMS tumors parallel data from UPS tumors showing similar myeloid cell profiles in primary and syngeneic models, but considerable model-specific differences in the T cell compartment.

### Gene expression of MHCI correlates with T cell infiltration

**Figure 7:**
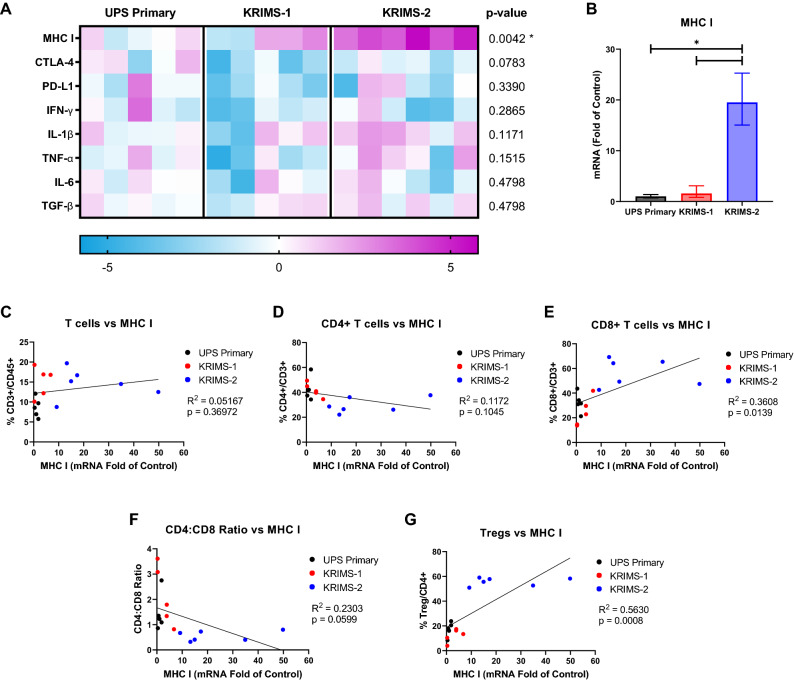
Expression of immunomodulatory genes in UPS tumor models. (**A**) Heat map of quantitative RT-PCR data analyzing gene expression of tumors matching those used for immunoprofiling in Figs. 3 and 4. Each column represents an individual tumor. Values are relative to the average expression of UPS primary tumors, with increased expression shown in pink and decreased expression shown in blue. Data analyzed by one-way ANOVA with a *p*-value < 0.05 denoted by a star. *n* = 5–6 tumors per group (**B**) *MHCI* expression is significantly elevated in KRIMS-2 tumors compared to UPS primary and KRIMS-1 tumors. Average mRNA fold change (2^-ΔΔCT^) ± SEM. (**C**–**G**) Correlation of *MHCI* expression and T cell populations using simple linear regression to analyze matching samples. There is strong positive correlation between *MHCI* levels and CD8 + T cells (**E**) and Tregs (**G**) in UPS tumors. R^2^ indicates goodness of fit. A *p*-value < 0.05 indicates a slope significantly different than zero. n = 16 mice per correlation analysis.

**Figure 8:**
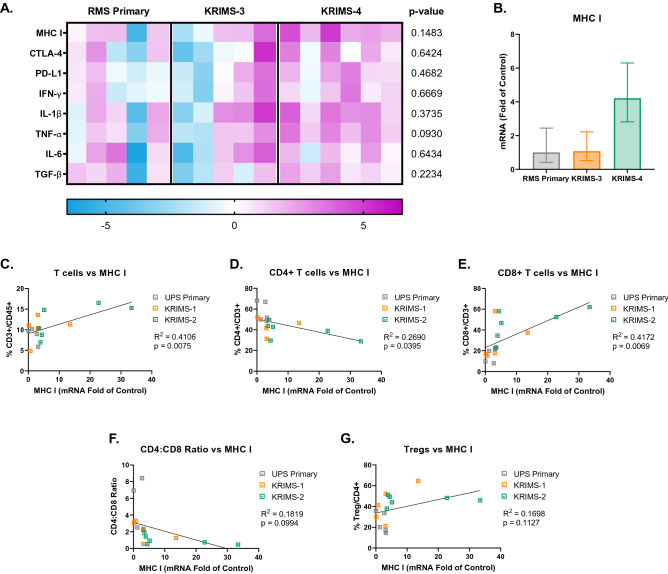
Expression of immunomodulatory genes in RMS tumor models. (**A**) Heat map of quantitative RT-PCR data analyzing gene expression of tumors matching those used for immunoprofiling in Figs. 5 and 6. Each column represents an individual tumor. Values are relative to the average expression of RMS primary tumors, with increased expression shown in pink and decreased expression shown in blue. Data analyzed by one-way ANOVA with a *p*-value < 0.05 denoted by a star. *n* = 5–6 tumors per group. (**B**) *MHCI* expression is elevated in KRIMS-4 tumors compared compared to RMS primary and KRIMS-3 tumors, though this is not statistically significant (*p* = 0.1483). Average mRNA fold change (2^-ΔΔCT^) ± SEM. (**C**–**G**) Correlation of *MHCI* expression and T cell populations using simple linear regression to analyze matching samples. There is strong positive correlation between *MHCI* has a strong positive correlation with CD3 + and CD8 + T cells (**C**, **E**), and a negative correlation with CD4 + T cells (**D**) and CD4:CD8 ratio (**F**). R^2^ indicates goodness of fit. A *p*-value < 0.05 indicates a slope significantly different than zero. n = 16 mice per correlation analysis.

To examine pathways that may contribute to the immune profiles of primary and syngeneic models, we performed gene expression analysis of whole tumor lysates from the same tumors profiled by flow cytometry^4^. Using quantitative RT-PCR, we evaluated expression of genes involved in tumor antigenicity, T cell activation, and cytokine signaling. This analysis identifies a broad range of gene expression across individual tumors, further underscoring the heterogeneity of each model (Figs. 7A and 8A). In UPS tumors, *MHCI* is the only gene that is statistically different between models (*p* = 0.0042, one-way ANOVA). Downregulation of MHC I presentation is a frequent mechanism for immune evasion in human tumors, resulting in reduced recognition by cytotoxic T cells^35,36^. Expression of *MHCI* is elevated in KRIMS-2 tumors, which also have the highest percentage of Tregs and CD8 + T cells among all models (Fig. 7A,B). We hypothesized that *MHCI* mRNA correlates with T infiltration across all UPS tumors. Indeed, there is a positive correlation between *MHCI* and levels of CD8 + T cells and Tregs in UPS, supporting a role for tumor antigenicity in T cell recruitment (Fig. 7C–G). In RMS tumors, we observe a similar enrichment of *MHCI* in KRIMS-4 tumors, which are the most T cell infiltrated model (*p* = 0.1483, one-way ANOVA, Fig. 8A,B). There is a strong correlation between *MHCI* expression and levels of multiple T cell subsets, including a positive correlation with CD3 + and CD8 + T cells, and a negative correlation with CD4 + T cells and the CD4:CD8 ratio (Fig. 8C–G).

To further explore differences in gene expression and T cell populations between models, we examined levels of immune checkpoint markers *CTLA4* and *PD-L1*. Expression of *CTLA4* is decreased in KRIMS-1 and KRIMS-3 tumors compared to their primary counterparts, while *PD-L1* is increased in KRIMS-4 tumors (Figs. 7A and 8A). *CTLA-4* negatively correlates with CD3 + T cells infiltration in UPS tumors and positively correlates with Treg levels in RMS tumors (Supplemental Figs. 5A–E and SF 6A–E). In RMS tumors, *PD-L1* expression has a strong positive correlation with Tregs and CD8 + T cells and is negatively correlated with CD4 + T cells (Supplemental Fig. 6F–J). In UPS models, there is no correlation between T cell populations and *PD-L1* levels (Supplemental Fig. 5F–J). We also examined expression of key cytokines involved in T cell activation, including proinflammatory molecules (*Tnfa*, *Ifng*, *IL1b)*, and immune-suppressive cytokines (*IL6* and *Tgfb*). No significant differences in cytokine gene expression were observed between the models, highlighting the importance of the link between *MHCI* expression and T cell infiltration in the primary and syngeneic tumor models.

## Discussion

Both GEMM and syngeneic murine tumor models are vital tools for cancer immunology; however, there is a need to better understand the critical differences in immune landscapes between these two approaches^37^. In the current study, we report the first side-by-side characterization of the immune composition of two primary soft-tissue sarcoma models and their corresponding syngeneic counterparts. All of these models have identical initiating mutations, occur in a similar orthotopic location, and arise within the same background strain. Comparing the immune profiles of UPS and RMS primary tumors, we find that while the level of total immune infiltration is similar between the two GEMMs, the proportion of CD4 + and CD8 + T cells differs significantly. From primary UPS and RMS models, we generated a series of syngeneic cell lines (KRIMS cells) that can be efficiently grown in immunocompetent mice. Here, we report the first comprehensive analysis of immune landscapes between primary and orthotopic syngeneic models. Through flow cytometry, we identify considerable differences across the T cell compartments of syngeneic tumors and their primary tumor counterparts. While levels of myeloid cells, NK cells, and B cells are relatively stable across all models, infiltration of Tregs, CD8 + T cells, and CD4:CD8 ratios are highly divergent between each model. We also observe immune variation across syngeneic tumors derived from the same primary model, with KRIMS-2 and KRIMS-4 displaying the strongest differences. Taken together, this analysis demonstrates the frequency of immune cell diversity across distinct syngeneic series.

In comparison to well-characterized syngeneic models of epithelial cancers, this study identifies key features of the immune landscape that are unique to sarcoma models. For example, TAMs are the predominant myeloid cell population in our UPS and RMS syngeneic tumors, whereas MDSCs are the most prevalent myeloid cell type in 4T1 breast and B16F10 melanoma models^5,6^. Levels of NK cells are very low in our sarcoma tumors, (< 2%), while NK cells are present at large numbers in RENCA and CT26 tumors (10–25%)^5^. The proportion of T cells infiltrating a tumor can depend upon mouse background strain, as T cells represent 12–21% of total CD45 + cells in syngeneic BALB/c models (CT26, RENCA, and 4T1), but only 4–8% of total CD45 + cells in syngeneic C57BL/6 models (MC38, LL/2, and B16F10)^5^. In our syngeneic sarcomas, T cells account for 9–15% of total immune cells, which is within the range of other T-cell rich tumor models. The majority of published syngeneic models of epithelial cancers report a CD4:CD8 ratio equal to or above 1^5–10^. In our study, 3 of the 4 of the syngeneic models have a CD4:CD8 ratio ≥ 1. However, KRIMS-2 tumors favor CD8 + T cell enrichment with a CD4:CD8 of 0.6 (Fig. 4E).

To explore potential drivers of altered T cell infiltration, we performed quantitative RT-PCR analysis of the same tumors used in flow analysis. We identified a strong correlation between *MHCI* expression and CD8 + T cell infiltration in both UPS and RMS models. *MHCI* downregulation is a common strategy that helps tumors avoid recognition by T cells. An estimated 40–90% of human tumors actively downregulate MHC I, which correlates with worse prognosis^35,36^. Another potential feature impacting immune infiltration is tumor growth kinetics. For example, the KRIMS-4 model displays increased tumor latency and higher levels CD8 + T cells and Tregs compared to other RMS models. Such data further underscores the necessity for careful characterization of the tumor microenvironment in the development of immune-oncology models^37^. To increase the translatability of preclinical cancer immunology studies, scientists should leverage the strengths of multiple models, such as transplanted tumor fragments or multiple syngeneic tumors within a series^38^.

Our data identify several similarities between the immune profiles of patient sarcomas and the mouse models described in this study. Sarcomas are not traditionally considered strongly immunogenic tumors, as patient sarcomas contain less tumor-infiltrating lymphocytes than other solid tumors, including renal cell carcinoma and melanoma^39^. In comparison to mouse models of epithelial tumors, our GEMM and syngeneic models have lower levels of immune infiltration, with CD45 + cells comprising only 10–20% of live cells in these tumors. In a large TCGA analysis of 206 patient samples across 6 different sarcoma types, UPS tumors have one of the highest median macrophage scores^40^. This macrophage predominance in UPS was confirmed in an immunohistochemical analysis of 1,242 sarcoma samples^41^. High numbers of mature macrophages are also common in RMS samples and correlate with improved survival^42^. In our UPS and RMS mouse models, TAMs represent 53–65% of immune infiltrate. In contrast, TAMs comprise only 5–19% of immune cells in commonly-used syngeneic models of epithelial cancers^5^. In comparison to other adult sarcomas, UPS has the highest levels of TCR clonality, T cell infiltration, and PD-1 expression, although these levels are still low in relationship to epithelial tumors^40,43^. This could be due to UPS having a higher somatic mutational burden than other STS types^40^. Analysis of patient RMS samples identifies low infiltration of CD3 + T cells, CD4 + T cells and Tregs compared with other epithelial tumors^42^. In the syngeneic mouse models of sarcoma, we identify modest levels of T cell infiltration (10–15%) that fall in midway across the range for models of epithelial tumors (4% to 21%).

These findings are of particular interest given the disappointing results of immunotherapy trials in STS^44–48^. In a phase II trial for metastatic sarcoma with nivolumab (PD-1 inhibitor) in combination with ipilimumab (CTLA-4 inhibitor), only 16% (6/38) of patients achieved a confirmed objective response^48^. UPS was the predominant sarcoma type among responding patients, although patients with other subtypes did respond^48^. Similar results were seen in a multicenter phase II study of pembrolizumab (SARC028), with 40% of UPS cases showing responses^44,46^. However, PD-L1 overexpression predicts poor overall survival in multiple sarcoma types, suggesting a role for tumor microenvironment-induced T cell suppression in sarcomas^49^. Other groups have identified B cell-rich tertiary lymphoid structures in sarcomas that respond well to immunotherapy^50^. In our primary and syngeneic models, both UPS and RMS tumors contain similar levels of B cell and total T cell infiltration, suggesting that RMS may respond to immunotherapy at similar rates as UPS. However, relatively few immunotherapy trials have been conducted in RMS due to the challenges of immunotherapy in pediatric patients^51,52^. Current clinical trials are attempting to increase the efficacy of immunotherapy in UPS and other responding sarcomas by combining checkpoint blockade with chemotherapeutic agents (NCT02888665) or radiation therapy (NCT03092323). Since both chemotherapy and radiotherapy can increase production of immune-stimulatory factors in cancer cells, a main objective of these approaches is to increase intratumoral immune cell recruitment^53,54^. With the continued challenges of immunotherapy response in many cancers, our study highlights how an expanded knowledge of the immune landscape in primary and syngeneic tumors can guide continued translational research.

## Methods

### Mice

All animal experiments were performed in accordance with protocols approved by IACUC at the University of Iowa. The LSL-Kras^G12D^, p53^Flox/Flox^ (KP) and Pax7-CreER; KP (P7KP) sarcoma models have been previously described^27–30^. For the UPS model in KP mice, 25 µL of Ad-Cre (University of Iowa Viral Vector Core, Iowa City, IA) was mixed with 3 µL calcium chloride (2 M) and 600 µL DMEM (Gibco, 11965-092). Following a 15-min incubation at room temperature, 50 µL of the mixture was injected into the gastrocnemius muscle. For the RMS model in P7KP mice, animals were injected with 50 µL of 4-hydroxytamoxifen (10 mM in DMSO, Sigma-Aldrich, H7904) in the gastrocnemius muscle. Tumors were measured by digital caliper three times weekly after tumors reached 200–285 mm^3^, and volume was calculated using the formula V = (π × *L* × *W* × *H*)/6, with *L*, *W*, and *H* representing the length, width, and height of the tumor in mm, respectively. Tumors outside of specified volume range at the time of detection were excluded from the study. All mice were maintained in the Dodd lab colony. Male and female mice over 7 weeks old were used for all studies.

### Derivation of cell lines

Terminal tumor tissue was dissected with forceps and surgical scissors, washed in 5 mL PBS in a 6-well plate, then finely minced with surgical scissors. 5 mL of Dissociation Buffer [Collagenase Type IV (700 units/mL, Gibco, 17104-019) and dispase (65 mg/mL, Gibco, 17105-041)] in PBS was added to each well. Plates were incubated for one hour at 37 °C on an orbital shaker and transferred to a tissue culture hood. Dissociated tissue was passed through a sterile 70 µM cell strainer (Fisherbrand, 22363548) into a 50 mL conical vial using a 10 mL serological pipette and the plunger from a 1 mL syringe (BD, 309628). Cell strainers were washed with 25 mL sterile PBS into corresponding conical vials. Cell suspensions were centrifuged, and cell pellets were resuspended and plated in DMEM (Gibco, 11965-092). Cells were grown in 10 cm dishes maintained in DMEM media containing 10% FBS, 1% penicillin–streptomycin (Gibco, 15140-122) and 1% sodium pyruvate (Gibco, 11360-070). When 90% confluency was reached, 15–35% of cells were passaged into a new dish. After a minimum of 10 passages, cells were frozen down to be used for subsequent analysis. For in vitro proliferation assays, cells were seeded in 96-well plates, and growth was measured at 0, 24, 48, and 72 h by resazurin cell viability assay (Sigma, R7017).

### Syngeneic allografts

All cells were ~ 90% confluent on the day of injection. Cells were trypsinized, washed, and resuspended in sterile PBS containing calcium chloride and magnesium chloride. Mice maintained on a 129/SvJae background were injected with 50 µL of cell suspension in the left gastrocnemius muscle using a 31G needle. For UPS allografts, mice were injected with KRIMS-1 (1 × 10^6^ cells) or KRIMS-2 (2.5 × 10^5^ cells). For RMS allografts, mice were injected with KRIMS-3 (2.5 × 10^5^ cells) or KRIMS-4 (0.5–1 × 10^5^ cells). After reaching a volume of 200–285 mm^3^, tumors were measured three times weekly by digital caliper.

### Histological analysis

Terminal tumor tissue was stored in 10% neutral buffered formalin for fixation and subsequent paraffin embedment. Formalin-fixed paraffin embedded tumors were sectioned and stained with hematoxylin (Vector Laboratories, H-3401) and eosin (Harleco, 200-12) to evaluate tissue morphology. Images were taken using an Olympus BX61 microscope (Olympus) at 40 × magnification, scale bars at 50 µm.

### Flow cytometry

When tumors reached terminal volume (~ 1200–1500 mm^3^), harvested tumors were washed with 5 mL PBS in a 6-well plate and finely minced with surgical scissors. 4.5 mL Collagenase Type IV (700 units/mL, Gibco, 17104-019) and 0.5 mL FBS were added to each well containing tumor tissue. Plates were incubated for one hour at 37 °C on an orbital shaker. Following incubation, dissociated tissue was passed through a 70 µM cell strainer into a 50 mL conical vial using a 10 mL serological pipette and the plunger from a 1 mL syringe. Cell strainers were washed with 25 mL PBS into corresponding conical vials. Cell suspensions were centrifuged, and cell pellets were resuspended in 2 mL ACK lysis buffer (Gibco, A1049201). After five minutes, 10 mL PBS were added, and samples transferred to 15 mL conical tubes and centrifuged at 500×g for 5 min. Cell pellets were resuspended in cell staining buffer (Biolegend, 420201). In a round-bottom 96-well plate, 50 µL aliquots of cell suspensions were incubated with Zombie Aqua Viability Dye (Biolegend, 77143) and anti-CD16/32 (clone 93, Biolegend) to block Fc receptors on ice. After a 10-min incubation, 50 µL of antibodies were added and incubated on ice for 30 min. Antibodies include: anti-CD45 BV605 (clone 30-F11, Biolegend), anti-CD11b PE (clone M1-70, Biolegend), anti-CD11c BV421 (clone N418, Biolegend), anti-CD3 PE-Cy7 (clone 145-2C11, Biolegend), anti-CD4 Alexa Fluor 700 (clone GK1.5, eBioscience), anti-CD8 PerCP/Cy5.5 (clone 53-6.7, Biolegend), anti-CD25 PE-Cy5 (clone PC61.5, Invitrogen), anti-NKp46 PerCP/Cy5.5 (clone 29A1.4, Biolegend), anti-F4/80 Alexa Fluor 488 (clone BM8, Biolegend), anti-B220 APC (clone RA3-6B2, Biolegend), and anti-I-A/I-E Alexa Fluor 700 (clone M5/114.15.2, Biolegend). Regulatory T cells were stained by anti-CD127 APC (clone SB/199, Biolegend) or anti-FoxP3 APC (clone FJK-16 s, Invitrogen) with the FoxP3/transcription factor staining buffer set (eBioscience, 00-5523-00). Stained cells were fixed (Biolegend, 420801) and stored in the dark at 4 °C for 24–48 h. Samples were analyzed with a BD LSR II flow cytometer. Data analysis was performed using FlowJo version 10.6.1 (Becton, Dickinson and Company). Fluorescence minus one (FMO) controls were used to set the boundary gates between positive and negative populations. Samples with < 55% viable cells were excluded from analysis.

### Quantitative RT-PCR

As previously published^4^, upon harvest, tumor tissue was stored in RNA Later (AM7020, Thermo Fisher Scientific) at − 20 °C. Tumors (*n* = 5–6 per group) were homogenized in liquid nitrogen and resuspended in Trizol for RNA extraction (15596018, Thermo Fisher Scientific). cDNA was synthesized from 1 ug of RNA using iScript (1708891, Bio-Rad). RT-qPCR was performed with Power-up Sybr Green 2 × Master Mix (A25778, Thermo Fisher Scientific) per the manufacturer’s instructions on an Applied Biosystems 7900HT instrument using the ΔΔC_T_ relative to 18 s expression (Genomics Division of the Iowa Institute of Human Genetics, University of Iowa). Primer sequences are listed in Supplementary Table 1. Gene expression correlations were performed using target gene mRNA fold change (2^−ΔΔCT^) values and immune cell population data from matching tumors.

### Statistics

Statistical analysis was performed using GraphPad Prism 8. Tumor growth data and immune profiles were analyzed using unpaired t tests with Welch’s correction (comparisons with two groups) or Welch’s ANOVA and Dunnett’s T3 multiple comparison test (comparisons with three or more groups). Data represent individual tumors with the mean ± SD. Gene expression data was analyzed by one-way ANOVA. Correlations between gene expression and immune cell populations were analyzed by simple linear regression.

### Study approval

All animal procedures for this study were approved by the Institutional Animal Care and Use Committee at University of Iowa, Iowa City, Iowa, USA and were carried out in accordance with ARRIVE guidelines.

## Supplementary Information

Supplementary Information.
