# Supplementary material for: Divergent immune landscapes of primary and syngeneic Kras-driven mouse tumor models
Source: Sci Rep. 2021 Jan 13;11:1098. doi: 10.1038/s41598-020-80216-1 (PMC7806664; doi:10.1038/s41598-020-80216-1)
Supplement: Supplementary file 1 — Supplementary Information. [file 41598_2020_80216_MOESM1_ESM.pdf]

Title: Divergent immune landscapes of primary and syngeneic Kras-driven mouse tumor models

Wade R. Gutierrez [1,2,3], Amanda Scherer [3,4], Gavin R. McGivney [1,3], Qierra R. Brockman [3,4], Vickie Knepper-Adrian [5], Emily A. Laverty [5], Grace A. Roughton [5], Rebecca D. Dodd [1,2,3,4,5]

[1] Cancer Biology Graduate Program, University of Iowa, Iowa City, Iowa

[2] Medical Scientist Training Program, University of Iowa, Iowa City, Iowa

[3] Holden Comprehensive Cancer Center, University of Iowa, Iowa City, Iowa

[4] Molecular Medicine Graduate Program, University of Iowa, Iowa City, Iowa

[5] Department of Internal Medicine, University of Iowa, Iowa City, Iowa

Corresponding author:

Rebecca D. Dodd

Carver College of Medicine, University of Iowa, 285 Newton Rd, 3269C CBRB, Iowa City, Iowa 52246

Tel: 319-335-4962, Email: rebecca-dodd@uiowa.edu

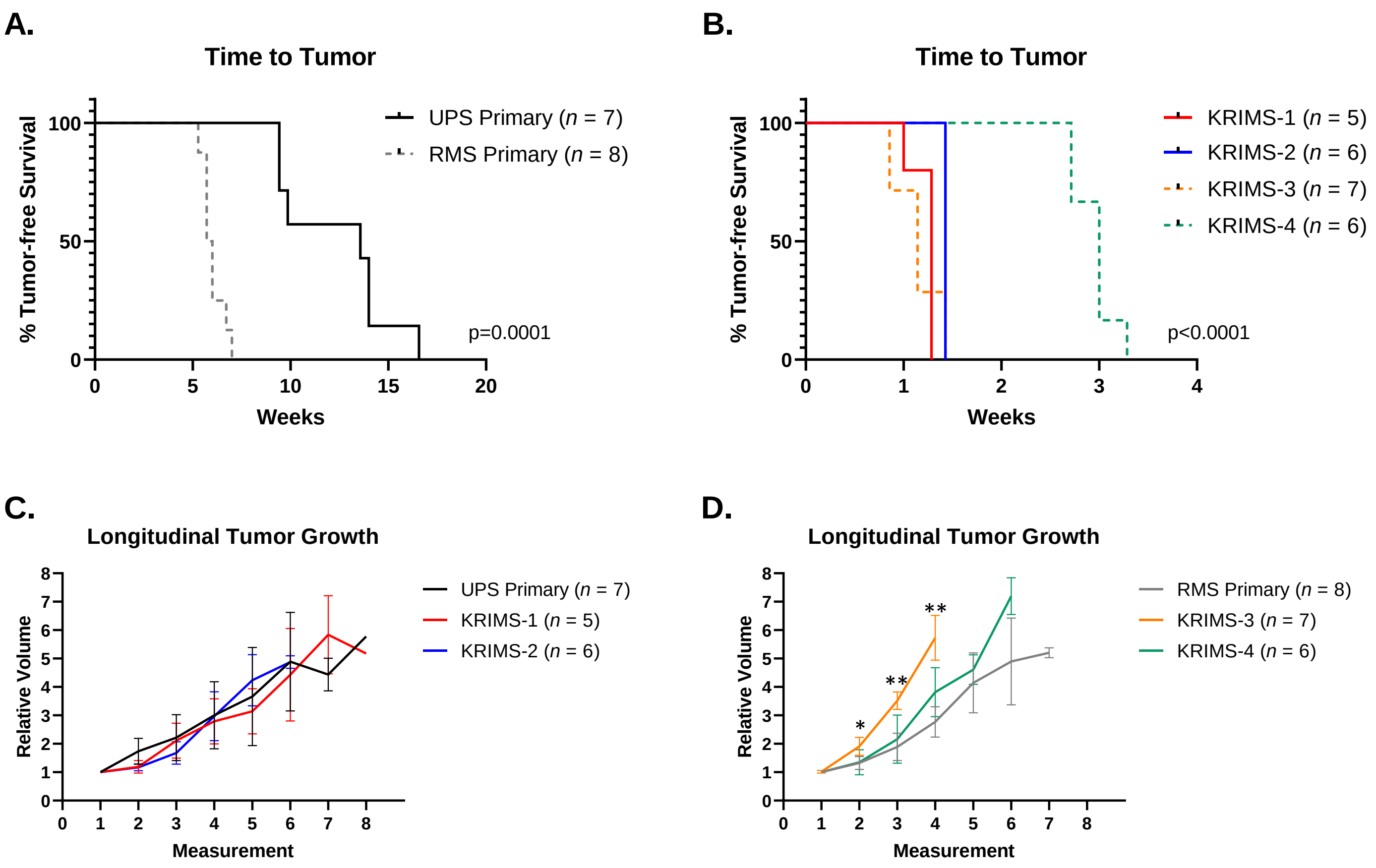

<u>Supplemental Figure 1: Tumor onset and longitudinal growth for primary and syngeneic models of sarcoma.</u> (A) Primary RMS tumors are detected on average 6.0 weeks after tumor initiation, while primary UPS tumors are detected on average 12.4 weeks after tumor initiation. (B) Following injection of KRIMS cells, syngeneic tumors developed within 1-3 weeks. After initiation, tumors were measured three times weekly. (C-D) Longitudinal growth analysis of UPS and RMS tumors. KRIMS-3 tumors were significantly larger than both RMS primary and KRIMS-4 tumors at later measurements (**) but were only larger than primary tumors at early measurements (*). Log-rank (Mantel-Cox) tests used to analyze (A-B). Welch's ANOVA and Dunnett's T3 multiple comparison test used to analyze data from individual measurements in (C-D). Data represent the mean ± SD. A p-value of < 0.05 is denoted by a star. n = 5-8 tumors per group.

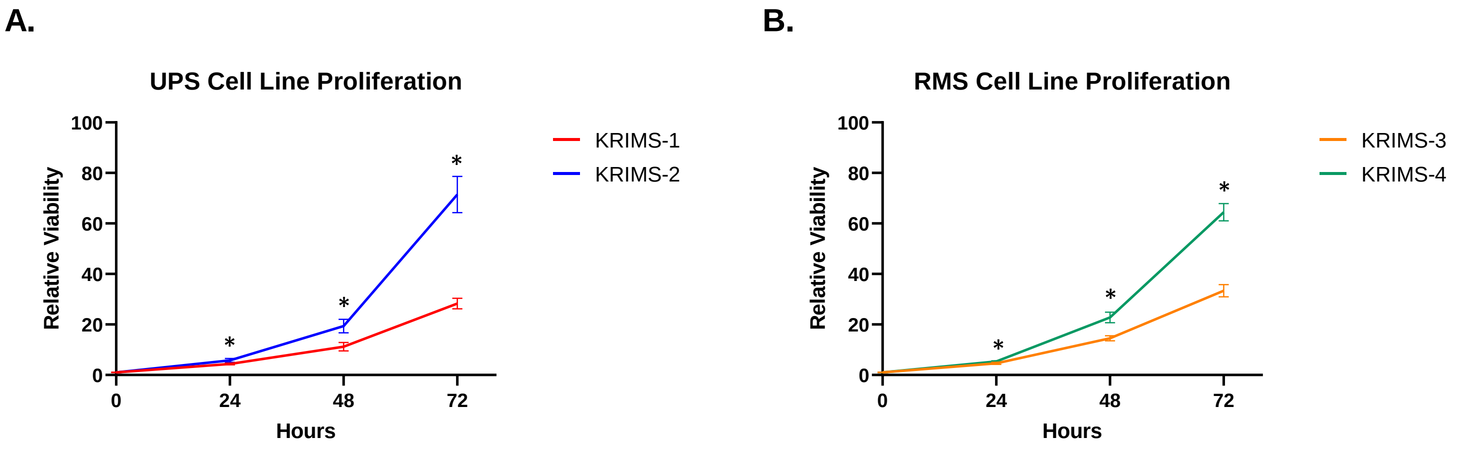

<u>Supplemental Figure 2: KRIMS cell line growth *in vitro.*</u> (A) Longitudinal growth of UPS cell lines, KRIMS-1 and KRIMS-2. Resazurin cell viability assays were performed at 0, 24, 48, and 72 hours. There was no difference in fluorescence at time 0. KRIMS-2 relative viability is significantly greater than KRIMS-1 at 24, 48, and 72 hours, indicating faster growth *in vitro*. (B) Longitudinal growth of RMS cell lines, KRIMS-3 and KRIMS-4. Resazurin cell viability assays were performed at 0, 24, 48, and 72 hours. There was no difference in fluorescence at time 0. KRIMS-4 relative viability is significantly greater than KRIMS-3 at 24, 48, and 72 hours, indicating faster growth *in vitro*. Unpaired T test with Welch's correction was used to analyze data at each timepoint. Data represent the mean ± SD of six technical replicates per cell line. A p-value of < 0.05 is denoted by a star.

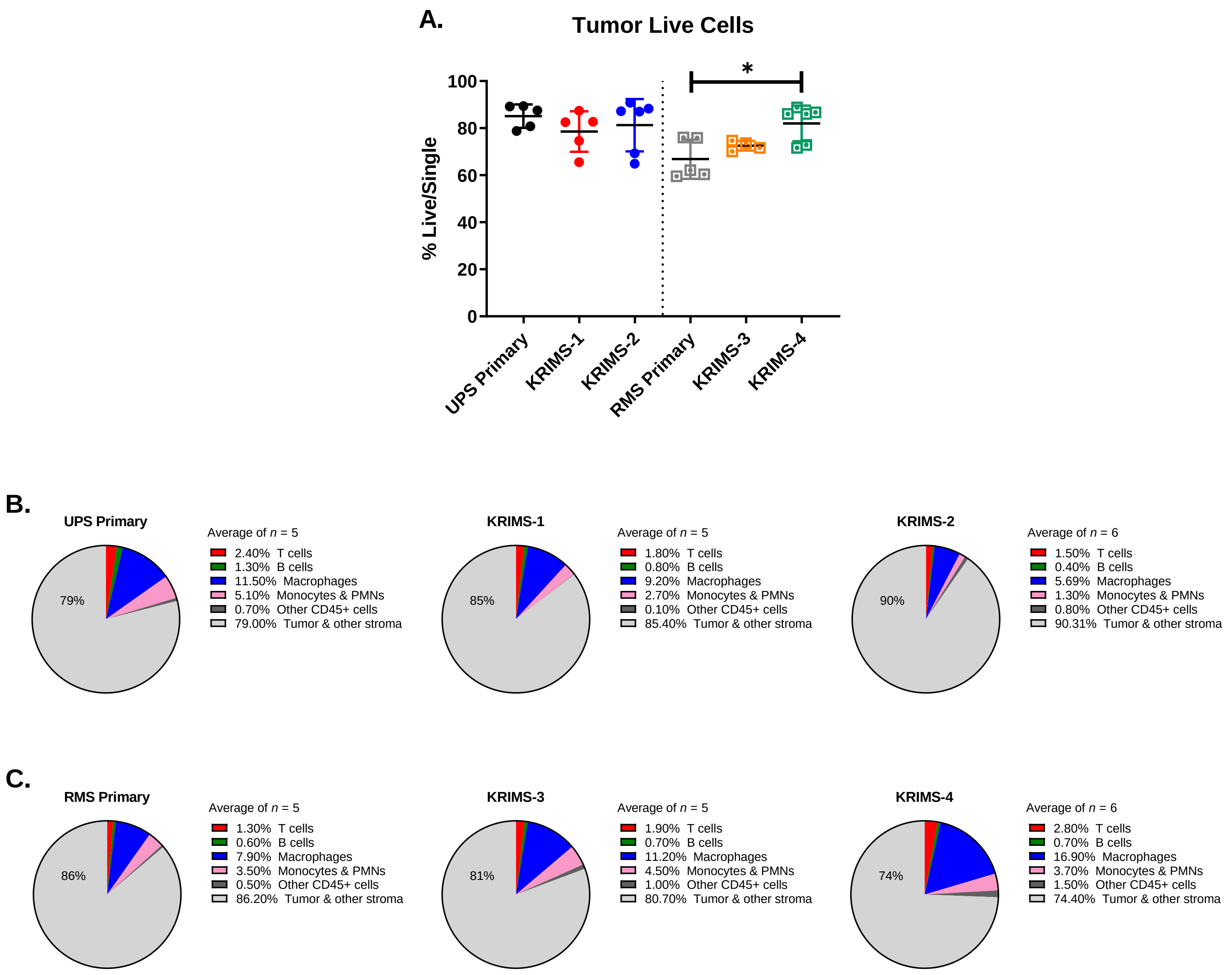

Supplemental Figure 3: Immune cell composition in tumor models, relative to live cells. Note that data reported in Figures 2-6 are relative to CD45+ cells (all immune cells), whereas this figure reports the same data relative to live cells (all viable cells within the tumor). (A) Percent of live cells detected in all tumors evaluated in this study. (B-C) Tumor immune cell composition as a percent of live cells. Values are an average of tumors evaluated in panel A. Welch's ANOVA and Dunnett's T3 multiple comparison test were used to analyze data in (A). Data represent individual tumors with the mean ± SD. A p-value < 0.05 is denoted by a star. n = 5-6 tumors for immune analysis.

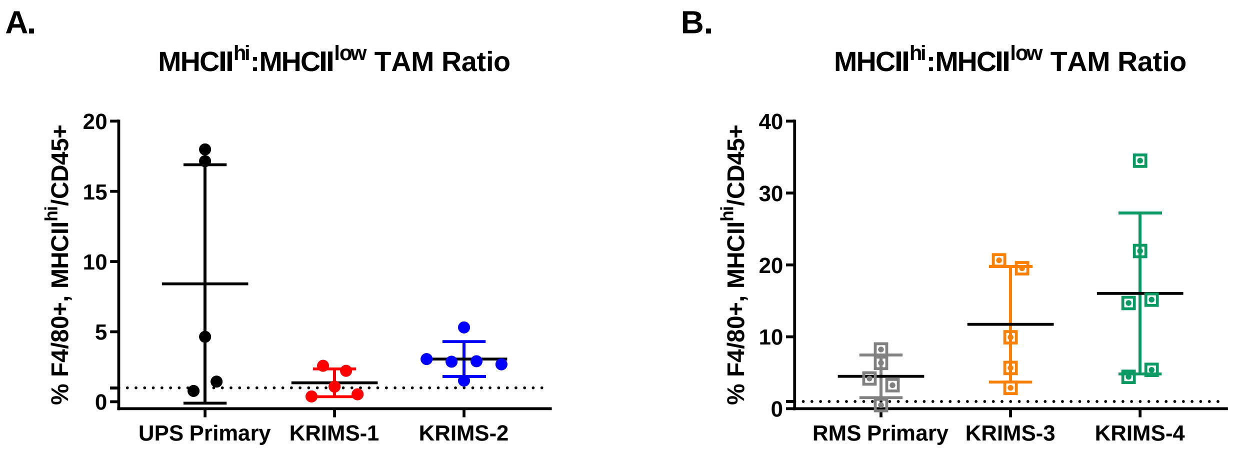

<u>Supplemental Figure 4: Ratio of MHCII^{hi} to MHCII^{low} tumor-associated macrophages.</u> Dotted line denotes a ratio equal to 1. MHCII^{hi} TAMs are more prevalent than MHCII^{low} TAMs in both UPS (A) and RMS (B) models, as shown by an MHCII^{hi}/MHCII^{low} ratio >1. Welch's ANOVA and Dunnett's T3 multiple comparison test were used to analyze data in. Data represent individual tumors with the mean ± SD. A p-value < 0.05 is denoted by a star. n = 5-6 tumors for immune analysis.

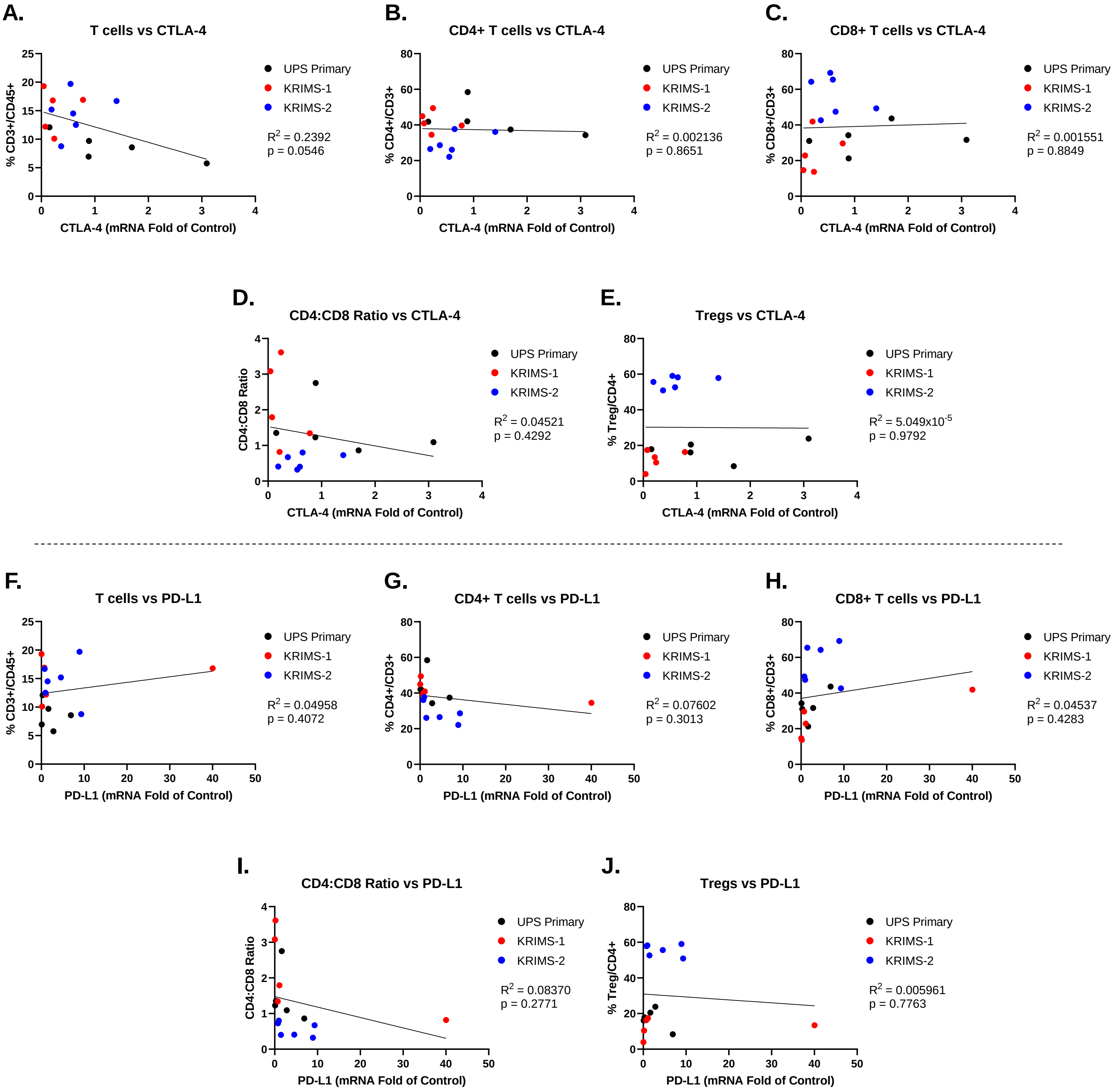

Supplemental Figure 5: Correlation of *CTLA4* and *PD-L1* expression with T cell levels in UPS tumors. (A-E) Correlation of *CTLA4* expression with T cell infiltration (A), CD4+ T cells (B), CD8+ T cells (C), CD4:CD8 Ratio (D), and Tregs (E). (F-J) Correlation of *PD-L1* expression with T cell infiltration (F), CD4+ T cells (G), CD8+ T cells (H), CD4:CD8 Ratio (I), and Tregs (J). Simple linear regression was used to analyze the correlation between gene expression and immune cell levels from paired samples. Data represent individual tumors. R^2 indicates goodness of fit. A p-value < 0.05 indicates a slope significantly different than zero. n = 16 mice per correlation analysis.

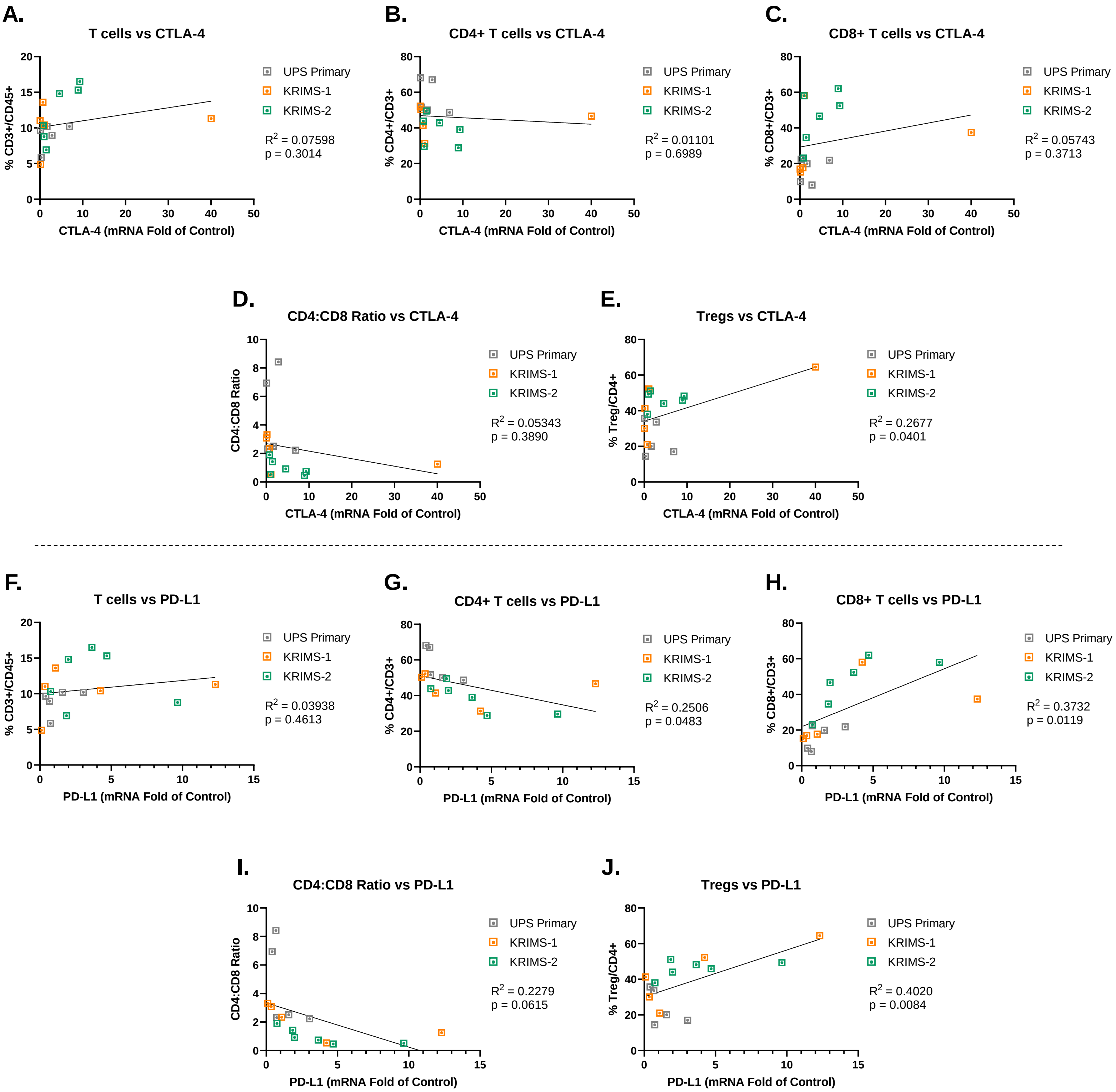

Supplemental Figure 6: Correlation of *CTLA4* and *PD-L1* expression with T cell levels in RMS tumors. (A-E) Correlation of *CTLA4* expression with T cell infiltration (A), CD4+ T cells (B), CD8+ T cells (C), CD4:CD8 Ratio (D), and Tregs (E). (F-J) Correlation of *PD-L1* expression with T cell infiltration (F), CD4+ T cells (G), CD8+ T cells (H), CD4:CD8 Ratio (I), and Tregs (J). Simple linear regression was used to analyze the correlation between gene expression and immune cell levels from paired samples. Data represent individual tumors. R^2 indicates goodness of fit. A p-value < 0.05 indicates a slope significantly different than zero. n = 16 mice per correlation analysis.

**Gene Name**	**Forward Primer Sequence**	**Reverse Primer Sequence**
MHC I (H2-Db)	AGTGGTGCTGCAGAGCATTACAA	GGTGACTTCACCTTTAGATCTGGG
CTLA-4	GCCTTCTAGGACTTGGCCTT	CACTGAAGGTTGGGTCACCT
PD-L1	GCTCCAAGGACTTGTACGTG	TAGTCTGAAGGGCAGCATTTC
IFN-γ	TCTGGAGGAACTGGCAAAAG	TTCAAAGACTTCAAAGAGTCTGAGG
IL-1β	TGGACCTTCCAGGATGAGGACA	GTTCATCTCGGAGCCTGTAGT
TNF-α	CTGTAGCCCACGTCGTAG	TTGAGATCCATGCCGTTG
IL-6	GAGGATACCACTCCCAACAGACC	AAGTGCATCATCGTTGTTCATACA
TGF-β	GGAGAGCCCTAGGATACCAAC	CAACCCAGGTCCTTCCTAAA
18s	GAGGCCCTGTAATTGGAATGA	GCAGCAACTTTAATATACGCTATTGG

Supplemental Table 1: List of PCR Primers.
